# “It's Not My Knee”: Understanding Ongoing Pain and Discomfort After Total Knee Replacement Through Re‐Embodiment

**DOI:** 10.1002/acr.24534

**Published:** 2022-03-26

**Authors:** Andrew Moore, Christopher Eccleston, Rachael Gooberman‐Hill

**Affiliations:** ^1^ University of Bristol Bristol UK; ^2^ University of Bath Bath UK; ^3^ National Institute for Health Research Bristol Biomedical Research Centre University Hospitals Bristol NHS Foundation Trust, and University of Bristol Bristol UK

## Abstract

**Objective:**

Up to 20% of people who undergo total knee replacement surgery have ongoing pain and discomfort. The aim of this study was to understand what role the concepts of embodiment (of both having a body and experiencing the world through one's body) and incorporation (integrating something into one's body) might have in understanding experiences of pain and discomfort after total knee replacement.

**Methods:**

We conducted semistructured interviews with 34 people who had received total knee replacement at either of 2 National Health Service hospitals in the UK, and who had chronic postsurgical pain (n = 34, ages 55–93 years). Data were audiorecorded, transcribed, and analyzed thematically.

**Results:**

Two main themes were identified: 1) when describing chronic postsurgical pain, some participants also described sensations of discomfort, including heaviness, numbness, pressure, and tightness associated with the prosthesis; 2) participants reported a lack of felt connection with and agency over their replaced knee, often describing it as alien or other, and lacked confidence in the knee.

**Conclusion:**

Participants’ experiences indicate that some people do not achieve full incorporation of the prosthesis. Our study emphasizes the importance of physicians treating patients as whole people and moving beyond clinical and procedural ideas of success. Our findings suggest that to optimize postoperative outcomes, rehabilitation must focus not only on strengthening the joint and promoting full recovery to tasks but on modifying a person's relationship to the new joint and managing sensations of otherness to achieve full incorporation of the joint or re‐embodiment.

## INTRODUCTION

Total knee replacement aims to reduce pain and restore function for people with advanced arthritis. Each year the number of knee replacements performed in the UK is approximately 102,000 and in the US 713,000 (1, 2). Total joint replacement has been described as one of the most successful orthopedic procedures (3, 4), but this idea of success is often based on technical parameters, implant survival, or procedural outcomes such as appearance on a radiograph. Decades of advancement in surgical technique and prosthesis technology have improved recovery outcomes, with fewer complications, greater pain relief, and longer implant survival, but approximately 20% of people still report ongoing pain and dissatisfaction after knee replacement (5, 6, 7, 8). This proportion is similar across the world, including in the UK, Sweden, and Canada (9). There is often no obvious procedural or mechanical explanation for poor outcomes and dissatisfaction after joint replacement, and Lape et al suggest that investigating the “embodied experience” of joint replacement, that is, further understanding the relationship between the altered body and the self, may provide further insight into why some people struggle with continued pain and functional limitations (5).

**SIGNIFICANCE & INNOVATIONS**

- This is the first empirical qualitative study to our knowledge to focus on understanding poor outcomes after knee replacement through an embodiment framework.
- For people with chronic postsurgical pain after total knee replacement, sensations of pain and discomfort may indicate a lack of effective incorporation of the prosthesis.
- We have identified a previously unreported sensation of limb tightness or squeezing, which needs further investigation.
- Future research should focus on identifying how best to manage sensations of otherness to modify a person's relationship to the new joint and to achieve full incorporation or re‐embodiment.

Typically, the assessment of patient‐reported outcomes after joint replacement focuses on functional outcome and pain relief as the main determinant of satisfaction (10). This narrow perspective is compounded by poor definitions of satisfaction after surgery (11), and there is little research on how and why some patients express dissatisfaction with joint replacement and what they are dissatisfied about. In their study of the meaning of satisfaction after hand surgery, Hudak et al found that some participants spoke about their hand as if it were an object separate from their self (“the hand was useless to me practically”) (12). This finding led the authors to suggest that a person's experience of dissatisfaction (or satisfaction) with treatment outcome was linked to their experience of their own body (12).

Research drawing on social and behavioral sciences has explored the experience of surgery that involves a prosthesis through the concepts of embodiment and incorporation (5, 12, 13, 14, 15, 16, 17). These concepts relate to the sense of connection with and agency over one's body and are grounded in the phenomenology of philosopher Maurice Merleau‐Ponty (18). Embodiment refers to the experience of the body as both subject and object, such that this idea impacts the way in which a person sees and interacts with the world, and vice versa (18, 19, 20). Embodiment provides a way of understanding how one experiences limits of possible action, a sense of control, and empowerment over physical action (18, 20). In a neutral state of embodiment, self and body are experienced as one, called the “lived body” (20). Illness, disease, and injury can force a disengagement of self from the body, or from 1 or more limbs, such that the body becomes “other” or alienated (21). How one comes to talk about the body as other is important to how one engages with rehabilitation. In chronic pain, for example, patients can come to discuss a joint, limb, or whole body as objectively other to the self, which is a risk for neglect, rehabilitation failure, and amputation (12, 22, 23, 24). Incorporation refers to the process of integrating something into one's body, either an object (e.g., a prosthesis) or a habit or skill (e.g., walking) (19). Evidence from studies on the incorporation of prosthetic limbs in amputees suggests that a strong sense of embodiment is one of the most crucial factors affecting functional recovery, and its absence may impede the efficient incorporation of a prosthesis (25, 26, 27).

Much of the previous literature on the incorporation of skeletal prostheses has focused on bodily extensions (13), or exoskeletal support such as wheelchairs or externally fitted prosthetic limbs (26). There is no examination of endoskeletal alteration such as internal prosthetic hip or knee joints, and little is known about how embodiment experiences relate to surgical outcomes and well‐being (5). De Preester suggests that there is no direct awareness of internal prostheses because unlike externally fitted limbs, they are nondetachable, and therefore less problematic (13). We challenge this notion, given that 20% of knee replacements result in ongoing pain and dissatisfaction. In this study, we use a discourse of embodiment as a lens through which to explore patient perceptions of ongoing pain and discomfort after knee replacement.

## PATIENTS AND METHODS

We conducted a qualitative study using semistructured interviews with people who had received a total knee replacement at 1 of 2 high‐volume National Health Service hospitals in the UK. The sample was purposive and diverse and designed to achieve data saturation, the point at which the collection of new data becomes unnecessary, which was reached at 34 participants (28).

After knee replacement, people may continue to experience improvements in pain and function up to 12 months postoperatively (29). To ensure that the sample included a diverse range of people whose pain and function was most likely to have stabilized, we approached individuals who had received their knee replacement between 12 months and 5 years previously. After ethical approval (15/WM/0469), a clinical team member identified potential participants from hospital patient lists and from a patient research cohort (the Oxford Musculoskeletal Biobank). Potential participants were sent an information pack and screening questionnaire about knee pain and health care use. Individuals who indicated a significant level of pain according to the Oxford Knee Score pain subscale (30), and who said their degree of contact with health professionals in the previous 12 months was “rare” or “never” were invited to participate. The sampling process was designed to enable inclusion of people with ongoing troublesome pain but who did not consult health care, reasons for which we have described elsewhere (31). Here we report on separate themes from the same study that focus on embodiment and discomfort. Therefore, this is not a secondary analysis, but further analysis of the primary data. All participants provided written informed consent to take part and for their anonymized quotations to be included in peer‐reviewed publications.

### Data collection

Interviews were conducted in participants’ homes by an experienced qualitative methodologist (AM), previously unknown to the participants. Interviews lasted between 32 and 105 minutes (mean 57 minutes) and took place from May 2016 to August 2018. A semistructured topic guide, designed in collaboration with the study's patient involvement group, was used to guide discussion about experiences of chronic pain after knee replacement. Topic areas included characteristics of pain, timing of pain onset and change over time, pain quality, pain duration and frequency, comorbidities, self‐management, and use of formal and informal health services. We had not planned to explore embodiment prior to interviewing, but by the third interview we noted that some participants described sensations of discomfort such as heaviness or numbness when discussing pain and some described their knee as “alien,” “foreign,” or “not part of” themselves. In response to these findings, the interviewer sought to elicit views about any such sensations in subsequent interviews, if this topic was not broached first by the participant.

### Data analysis

Interviews were audiorecorded, transcribed, anonymized, and uploaded to QSR NVivo 12 data management software (32). An inductive thematic analysis (33) was undertaken in which AM assigned codes and 2 other team members independently coded 4 transcripts, and developing codes were discussed, refined, and then applied across the data set. Relationships between codes were examined and codes grouped into themes. We have previously published findings from themes that relate to reasons for nonuse of health care services for chronic postsurgical pain (31); in this article we present findings relating to embodiment.

## RESULTS

A total of 34 people participated (18 women), all of whom had received a total knee replacement between 14 to 68 months before interview. The average age was 74 years (range 55–93 years). See Table 1 for participant characteristics.

**Table 1:** Participant characteristics (n = 34)

Characteristic	No.
Age group, years	
55–64	6
65–74	12
75–84	12
>85	4
Sex	
Male	16
Female	18
Time post‐knee replacement at interview, months	
12–24	7
25–36	11
37–48	13
>48	3

All participants described having pain, but 18 of the 34 individuals interviewed (53%) also described other discomforting sensations that are the focus of this study. Two main themes developed. When asked about the characteristics of postoperative pain, some participants additionally described their knee in terms of discomfort, including sensations of heaviness, numbness, pressure, and tightness. Participants also reported a lack of connection with and control over their replaced knee, often describing it as alien or foreign, which can be described as disembodiment. We present these findings with quotations from the interviews, so that we use the participants’ words to describe the theme. All names are pseudonyms.

### Sensations of discomfort

#### Heaviness and numbness

Some participants described their knee in terms of heaviness and numbness, to the point of discomfort: “Compared to what I had before I had the operation, it's not pain, it's discomfort…the only way I can describe it. As I say, it's 24/7 and it actually feels like your mouth feels after you've had your teeth out. That numbness and it's heavy” (Donald). He described how the feeling of heaviness impeded his movement: “My leg feels like it's made of lead. It gets heavy and I wouldn't say it's a sharp pain, but it's very uncomfortable, it's like walking with a lead band round your leg, you know, it's become very heavy.” Similarly, Peter described the sensation of heaviness as burdensome because he had to attend to his knee in order to sleep more comfortably: “I have to put a pillow between my legs to stop, cause it weighs a ton. Oh, it is heavy, very heavy. If I lie in bed, I either lie on one side, I cannot allow this leg to hit this one. I don't know why, but it just feels so heavy.”

Experiences of heaviness or lightness can be confusing and challenging. However, not all participants experienced the same sensation when asked about heaviness: “I heard somebody say that, but I don't find that” (Gwen).

#### Pressure and tightness

Other participants described a tightness or pressure around the knee that restricted movement, and which appeared to be different from descriptions of swelling or mechanical stiffness sometimes associated with knee replacement: “Yeah, it is different, it feels like someone is holding your knees, when you move, it's like someone is like, putting pressure there, when I move” (Brenda). Gwen also described a tightness around the knee that increased when she wore trousers: “I've noticed that I can't wear trousers for very long, because it feels like a tight band around it…I find it really uncomfortable to have anything tight around my knees.”

Peter also described feeling that he was encased: “It's like tightness, it's like being encased in a very tight skin. That's all the area contracted down there; everything is pushing on me.” What appears to be common in these experiences is that, unlike tumescence (the state of being swollen), the tightness is experienced as an external force, pushing on or holding the knee, rather than a mechanical tightness emanating from within the knee.

#### Sensations of disembodiment

Together with pain and sensations of discomfort, patients’ language demonstrated a separation of the affected limb from self. Participants described awareness of the inorganic nature of their prosthesis, which for some precluded any idea that the prosthesis could be fully accepted into the body: “It feels like it's someone else in me. [Is that how you feel about it?] Yeah, that's how I feel about it. Yes. It doesn't belong to me” (Phoebe). Celia rationalized that having a prosthesis would change a person's experience of their body: “When you've got a foreign object in you, it's never going to be the same again, is it? Never going to be like it was.” When asked about the ongoing pain in his knee, Tom explained why he thought it had not resolved, and suggested that he did not feel connected with his “alien” knee: “Well the best way when I think about it is to say it, I know it's not my knee. It's an alien knee in there. I don't really feel connected to it.” Wendy also objectified the prosthesis, as not only something apart from her lived body, but something beyond her control: “I think the physio tried her very best, but I think it's the knee itself probably…that knee just wouldn't do what it's told to do.”

#### A lack of connection

Linked to the experience of strange sensations and the idea of alienation from the knee, participants also described how a lack of felt connection to the knee meant their movement was no longer natural but required deliberate attention: “If I was to walk across there now and…because [of the dog] on the floor, whereas any normal person would walk along and step over him, I have to stop and think about stepping over him. My knee won't let me do that.” Brenda shared a similar experience: “I do trip a lot, because if there's something on the floor…if I think about it, I know I've got to lift that leg right over, but if I don't, I can't lift it enough.”

Tom described his attempt to overcome the sense of a lack of connection and how he made a conscious effort to focus on his replaced knee: “It doesn't feel like my knee any longer. You have to think about everything you do with it, whereas before it was sort of subconscious.”

### The unreliable body

There was a distinction between the sense of otherness that some participants felt, which suggests a disruption between body and self, and a lack of “conscious connection,” whereby some participants describe difficulty controlling their knee, which could without warning result in a fall: “I know it's not my knee. [Your real knee?] Yeah. [But how, what's that sensation like?] Like you're not connected to it fully. You know, like when I'm baking out there this morning, I stand up 20 minutes or so, and my knees will shoot forward. They'll do it independently…because it's not, it's not consciously connected to me. The best way I can describe it is it's not my knee” (Tom).

Tom associated the lack of feeling in his knee and the sense of disconnectedness as part of the same phenomenon, and like other participants, he talked about the prosthesis as an “it,” which being “inert” would let him down, if his focus wavered: “This one just…as I get more and more tired, that collapse happens more frequently. It will put me on the floor if I ignore it. And I've got to be really careful…I don't go out that often and I wouldn't fall every time, I go out, but I fall, you know, 2 or 3 times a week.”

Others also expressed concerns about falling and lacked confidence in their knee: “If I was in the garden working and I stepped over something, took a step over, I don't seem to have the confidence to be able to step back using this leg. I'm not confident enough to use that leg” (Harriet). “I always use the stick, I got to use a stick if we go out because I feel more confident. I haven't got the confidence to trust that knee because I'm frightened of falling, so terrified of falling” (Jerry).

### Intact limb as a prothesis: “It's part of me now.”

In contrast to those who struggled to feel a connection with their knee, others talked about the knee as a part of them, even despite sensations such as heaviness: “I would say a bit heavy, but numbness, no. Well I know it's a false knee, because you know up here, so. But I don't look upon it as not my knee. It's replaced my knee, and it's there to do a job for me…I don't look upon it as not…it's part of me now, yeah” (Desmond). Similarly, when it was suggested that some people did not feel like their knee was a part of them, Ada suggested otherwise: “Mine does. The only thing I can't do, I can't, if I kneel down, I find difficulty getting up.”

## DISCUSSION

While all participants in this study spoke of pain, our results also indicate that some struggled with additional discomfort relating to the prosthesis and experienced it as alien and “other” than the body, resulting in a lack of felt connection and confidence in the knee. Participants’ descriptions of otherness included pressure sensations, such as heaviness, which made moving the limb a conscious and effortful action. Such sensations are often vascular in origin but normally trigger worry, worry perhaps exacerbated by the idea of material being added, and now invisible, to one's body. We may also have identified a previously unreported sensation of limb squeezing, different from swelling, which needs further investigation. While swelling is associated with sensations of bursting (34), this feeling appears to be experienced as an external force, acting upon, rather than emanating from the knee. Ott and Maihöfner describe pressing and constricting in relation to symptoms of complex regional pain syndrome (CRPS) (35), and the American National Institute of Neurological Disorders and Stroke also lists symptoms associated with CRPS, including “as if someone were squeezing the affected limb” (36).

Our findings extend those of Gustafsson et al, who found that some patients with knee replacement experienced their body as unreliable, leading to falls that caused ongoing anxiety (22). Pain and discomfort appear to be linked to a sense that the prosthesis is “foreign” or “alien” and participants expressed a disconnection from the knee that meant they struggled to perform everyday tasks or risked falling. Similar to Hudak et al (14), this disconnection was reflected in participants’ language. Frequent reference to the knee as “it” implied an inertness that precluded the possibility of connection. These separation experiences are not neurologic, but rather expressions of a lost sense of ownership (the feeling of “mineness” experienced toward a body part), and lost agency (the experience of initiating and controlling an action) over the extremity (37).

The prosthesis remained in the conscious awareness of participants during movement, remaining quasi‐transparent (19), such that it did not withdraw from experience entirely, but participants experienced movement through the knee as effortful or awkward. This finding suggests that full incorporation had not been achieved. Murray finds that user rejection of a prosthetic limb can be negated by perseverance in use, until negative experiences give way to a more natural unconscious use (16). However, if individuals have spent years with chronic joint pain, any unconscious use may be a distant memory. As Lape et al (5) suggest, presurgical chronic pain, instability, and untrustworthiness might continue to influence incorporation of the prosthesis afterwards. However, not all participants struggled with re‐embodiment. Patients Desmond and Ada talked about a sense of ownership, perceived the knee as part of themselves, and focused on the functional possibilities the knee afforded them, despite discomforting sensations. Future research might focus on what can be learned from such experiences and attitudes.

In terms of optimizing postoperative outcomes, our study suggests that the interest for rehabilitation becomes not only strengthening the joint and promoting full recovery to tasks, but also modifying a person's relationship with the new joint to achieve full incorporation or re‐embodiment. Multidisciplinary rehabilitation programs for other conditions, including CRPS, include a range of strategies to increase re‐embodiment and improve body perception disturbances (38). Enhanced or virtual reality interventions show promise in improving body perceptions, function, and proprioception in musculoskeletal conditions, including knee replacement (39, 40, 41). Evidence from studies with amputees suggests that multisensory interventions that take advantage of brain plasticity to remap neural pathways may improve embodiment of assistive devices (26), and more collaborative research with fields such as psychoprosthetics and neurorehabilitation may help to improve outcomes after surgery (15, 25, 27, 42, 43).

This study is the first empirical qualitative study that we know of to focus on knee replacement through an embodiment framework. Previous literature has focused on the disruption between the body and self after surgery (14, 22, 24), which contrasts with our work that focuses on incorporation of the prosthesis. We also identified new aspects of sensation (squeezing) that need to be understood more fully to provide better knowledge of embodiment issues after knee replacement. This more complete understanding could inform development of outcome assessments that more adequately capture these aspects of knee replacement. Future work should explore whether sensations of discomfort and issues of re‐embodiment are present in people who do not report ongoing pain after knee replacement. Studies might also further examine the relationship between sensations of discomfort and pain and incorporation of various types of joint replacement prosthesis, such as the hip or shoulder.

A limitation of the study is that we did not collect information on reasons for nonparticipation, but the diverse characteristics of the sample and achievement of data saturation (28) means that we are confident that the findings are transferable to other people who received knee replacement in the UK. We also recognize that health systems vary between contexts and countries, but a study in Sweden also found that some patients lacked confidence in their replaced knee because of a lack of embodied connection (24). Another possible limitation is the risk that participants may give socially desirable accounts rather than their real perspectives (44). However, we feel that in‐depth questioning revealed authentic accounts and the interviewer's nonclinical background, and identification of participants who did not experience disembodiment issues, mitigate against this possibility.

Our research extends a theoretical grounding for understanding re‐embodiment after knee replacement, which can be used to inform the development of appropriate outcome measures and rehabilitative interventions. Our study emphasizes the importance of physicians treating patients as whole people and moving beyond clinical and procedural ideas of success. A more holistic assessment of the barriers to optimal improvement after surgery is needed. In addition to procedural influences (surgical skill, materials, and technique), we should consider patients’ preoperative expectations of surgery and recovery, including expectations about the prosthesis, health coping styles, social support, and barriers to physical rehabilitation (e.g., housing, work demands, etc.). Careful communication and listening to patients who experience functional difficulties with the prosthesis may reveal a struggle to incorporate the prosthesis. Discussing how the prosthesis feels different, and helping people to see it as part of their body, may reduce feelings of otherness and increase a sense of ownership and agency (5, 37).

Replacing any part of the body with something inanimate may have considerable impact, but the goal of medicine is to restore health as far as possible. Fostering an embodiment approach could help clinicians and researchers, in partnership with patients, to identify rehabilitative strategies that might facilitate more successful incorporation of prostheses. Our focus should not be on the absence or loss of embodiment, but on employing a multidisciplinary approach to using the concept to guide the development of pre‐rehabilitative strategies and appropriate outcome measures.

## AUTHOR CONTRIBUTIONS

All authors were involved in drafting the article or revising it critically for important intellectual content, and all authors approved the final version to be submitted for publication. Dr. Moore had full access to all of the data in the study and takes responsibility for the integrity of the data and the accuracy of the data analysis.

### Study conception and design

Moore, Eccleston, Gooberman‐Hill.

### Acquisition of data

Moore.

### Analysis and interpretation of data

Moore, Eccleston, Gooberman‐Hill.
